# Nomenclature of Diseases, (Woodworth)

**Published:** 1874-09

**Authors:** 


NOMENCLATURE OF DISEASES: Prepared for the use of the medical officers of the United Stated Marine Hospital Service, by the Supervising Surgeon (John M. Woodworth, M.D.) Being the classification and English-Latin terminology of the provisional nomenclature of the Royal College of Physicians, London. Washington: Government Printing Office. 1874. Octavo; cloth; pp. 210.

This very complete and systematic classification of diseases will be welcomed and prized by the favored few, outside the Government Hospitals, who may be so fortunate as to receive it, and who will owe a debt of gratitude to Dr. Woodworth for placing it within their reach.

---

THE TONER LECTURES: Instituted to encourage the discovery of new truths for the advancement of medicine. Lecture 3d.—On Strain and Over-action of the Heart. By J. M. Da Costa, M.D., Professor of Practice of Medicine in Jefferson Medical College, Philadelphia; Physician to the Pennsylvania Hospital, etc. Delivered May 14th, 1874. Washington: Smithsonian Institution, August, 1874. Pamphlet; pp. 28.

---

EXPERIMENTAL RESEARCHES ON THE PHYSIOLOGICAL AND THERAPEUTIC ACTION OF THE PHOSPHATE OF LIME. By L. Dusart, Ex-Interne des Hospitaux de Paris. Second Edition. Paris: 113 Faubourg St. Honore. 12mo; paper; pp. 168. From E. Fougera & Co., New York.

---

THE RELATION OF MEDICAL SOCIETIES TO PROGRESS IN SCIENCE: Inaugural Address of the President of the Medical Society of the county of Kings, New York. Alex. J. C. Skene, M.D., June 16th, 1874.

---

THE ADVANTAGE OF TORSION AS A MEANS OF ARRESTING HEMORRHAGE: Read before the Dallas city Medical Society, Dallas, Texas. By E. T. Easley, A.M., M.D., member of American Medical Association, author of a prize essay on the circumstances modifying the mortality after amputations, etc. Dallas, Texas 1874. Pamphlet; pp. 8.

